# Acute effects of high intensity interval training versus moderate intensity continuous training on haemostasis in patients with coronary artery disease

**DOI:** 10.1038/s41598-024-52521-6

**Published:** 2024-01-23

**Authors:** Daniel Košuta, Marko Novaković, Mojca Božič Mijovski, Borut Jug

**Affiliations:** 1 Faculty of Medicine, University of Ljubljana, Ljubljana, Slovenia; 2 Department of Vascular Diseases, University Medical Centre Ljubljana, Zaloška Cesta 7, 1000 Ljubljana, Slovenia; 3 Faculty of Pharmacy, University of Ljubljana, Ljubljana, Slovenia

**Keywords:** Coronary artery disease and stable angina, Biomarkers, Vascular diseases

## Abstract

Exercise training is associated with an acute net increase in coagulation, which may increase the risk of atherothrombosis in coronary artery disease (CAD) patients. We sought to compare the acute haemostatic effects of a bout of moderate-intensity continuous (MICT) and high-intensity interval training (HIIT) in patients with CAD. Patients after a recent myocardial infarction were randomized into a HIIT or MICT session of exercise training on a stationary bike. Blood was sampled at baseline, after the exercise bout and after a one-hour resting period. We measured overall haemostatic potential (OHP), overall coagulation potential (OCP), fibrinogen, D-dimer and von Willebrand factor (vWF) and calculated overall fibrinolytic potential (OFP). Linear mixed models for repeated measures were constructed to assess the treatment effect. A total of 117 patients were included. OCP, OHP, fibrinogen, D-dimer and vWF significantly increased after exercise and returned to baseline after a one-hour rest, OFP decreased after exercise and returned to baseline levels after a one-hour rest. Linear mixed models showed a significant difference between HIIT and MICT in fibrinogen (p 0.043) and D-dimer (p 0.042). Our study has shown that an exercise bout is associated with a transient procoagulant state in patients with CAD, with similar exercise-induced haemostatic changes for HIIT and MICT.

## Introduction

Cardiac rehabilitation is a multidisciplinary intervention comprising nutrition counselling, risk factors modification, education, psychosocial support and exercise training. Participation in cardiac rehabilitation is highly recommended for patients with coronary artery disease (CAD) as it yields a significant reduction in recurrent atherosclerotic cardiovascular (CV) events, an improvement in exercise capacity and an improvement in quality of life^1–5^. Cardiac rehabilitation is predominantly exercise based – most facets of cardiac rehabilitation are introduced through an exercise training program, providing adequate structure and supervision for effective and safe delivery of exercise-induced cardiovascular benefits^1^. Most evidence based cardiac rehabilitation programmes for patients with CAD provide endurance training either through moderate intensity continuous protocols (MICT) or high intensity interval protocols (HIIT)^6,7^. MICT (e.g. 30 min of continuous bicycling at 50–75% peak oxygen uptake [VO_2peak_]) is traditionally more established and perceived as safer, whereas HIIT (e.g. alternating intervals of 2–5 min of high intensity at ≥ 85% VO_2peak_ and low-intensity at 50–60 VO_2peak_) is possibly more effective (i.e., allowing faster and/or larger improvements in the level of fitness)^8–13^.

In patients with established CAD, the safety-versus-effectiveness of exercise training is a paramount parameter of exercise training prescription. Acute bouts of exercise result in seemingly unfavourable changes, such as transient vascular dysfunction and increased inflammatory markers, reactive oxygen species, transient vascular dysfunction, and increased coagulation^14–17^. Hypercoagulability represents one of the main pathophysiologic findings in venous thromboembolic events, however lately it has been shown that it has a pivotal role in arterial thrombotic events and in the manifestation CAD in particular^18–20^. Coagulation biomarkers such as fibrinogen, D-dimer and von Willebrand factor have been shown to be elevated in patients with CAD^21–23^, with a strong predictive value for adverse cardiovascular events and even mortality^23–25^. Exercise-induced hypercoagulability is especially concerning in patients with CAD, wherein haemostatic derangements may play a central role in the transition from stable atherosclerotic vascular diseases to life-threatening atherothrombotic complications, such as myocardial infarction and stroke^26–31^. Previous studies in healthy individuals have shown an acute increase in factor VIII-mediated hypercoagulability during bouts of exercise, with levels of, and balance between, coagulation and fibrinolysis strongly depending on training intensity and duration^32^. Conversely, studies with other haemostatic factors yielded inconclusive results^33^. To the best of our knowledge, no study to date compared the haemostatic effects of a bout of MICT with a bout of HIIT (i.e., a bout of exercise as experienced in real life by untrained patients with CAD).

Haemostatic derangements—including thrombotic occlusions in patients with CAD and exercise-induced hypercoagulability—represent complex processes, which may not be captured by measuring isolated coagulation or fibrinolysis biomarkers. In this regard, global haemostatic assays may provide more comprehensive appraisal of the coagulation and fibrinolysis interplay^34^. The overall haemostatic potential (OHP) is one such global haemostatic assay, which allows in vivo measurement of coagulation (i.e., fibrin formation, by adding thrombin) and fibrinolysis (i.e., fibrin degradation, by adding tissue plasminogen activator). OHP has been validated in healthy individuals and in a number of diseases, including CAD, wherein suggested increased coagulation and an impaired fibrinolysis^35–39^.

We hypothesized that a bout of exercise would increase coagulation and decrease fibrinolysis (as assessed by OHP) and the magnitude of effect would be greater in HIIT than MICT.

In the present study, we sought to compare the acute haemostatic effects of traditional MICT and increasingly popular HIIT in patients with CAD.

## Results

A total of 126 patients were screened for eligibility, 9 were excluded (5 refused to participate, 4 had missing data). A total of 117 patients were included, 59 were randomized in the HIIT group, 58 in the MICT group. We obtained a total of 447 adequate samples for analysis (3 were of inadequate quality for analysis in HIIT group at time point T0, 2 were of inadequate quality for analysis in HIIT group at time point T1, 2 were of inadequate quality for analysis in HIIT group at time point T2, 2 were of inadequate quality for analysis in MICT group at time point T0, 3 were of inadequate quality for analysis in MICT group at time point T1, 2 were of inadequate quality for analysis in MICT group at time point T2). All other measurements were performed and analysed for all patients at all prespecified timepoints as per protocol. All participants achieved the required heart rate (HR) (median achieved HR in the MICT group 128 bpm vs median achieved HR during in the HIIT group 131 bpm; p 0.399). No adverse events were registrated.

**Figure 1:**
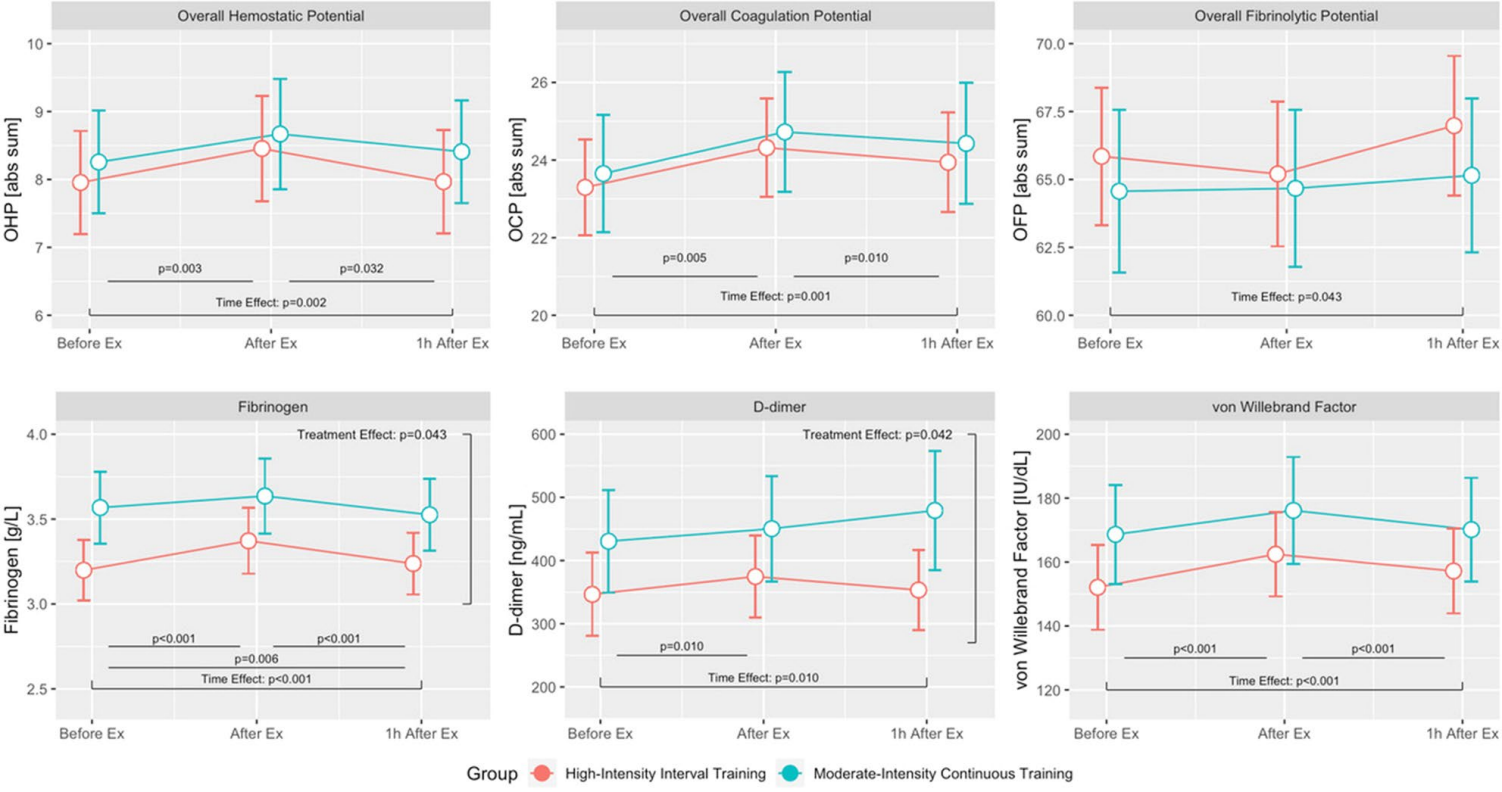
Time effect of coagulation markers. Change from baseline over time, estimates for overall coagulation potential (OCP), overall haemostatic potential (OHP), overall fibrinolytic potential (OFP), (D)—fibrinogen, (E)—D-dimer, (F)—von Willebrand factor. *Ex* exercise.

Baseline characteristics, risk factors presence and medical therapy are listed in Table 1. All patients at the time of event underwent coronary angiography with primary coronary intervention when appropriate, no patient underwent bypass grafting. Mean time from PCI to exercise testing did not differ between groups (96.6 ± 40 days for HIIT and 96.7 ± 38 days for MICT, p 0.993). A comparison between men and women in baseline characteristics is presented in Table 2. No patient had a history of atherothrombotic events or previous myocardial infarctions, no patients had sign or symptoms of overt heart failure. We measured OCP, OHP, OFP, fibrinogen, D-dimer, von Willebrand factor before the exercise bout, immediately after the exercise bout and after a one-hour rest (Table 3). There was a significant increase in OCP, OHP, fibrinogen, D-dimer and von Willebrand factor and a significant decrease in OFP after exercise, the values of all measured biomarkers returned to baseline after a one-hour rest. To compare the effect of HIIT and MICT on coagulation parameters a linear mixed model for repeated measurements was constructed, implementing subjects as random effects, repeated measurements and exercise type as fixed parameters and gender and age as covariates. Time (before exercise bout, immediately after exercise bout and one hour after exercise bout) was found to be significant for all measurements (Fig. 1). Time*group interaction (i.e. treatment effect) was significant for fibrinogen and D-dimer, but not for OCP, OHP, OFP and von Willebrand factor (Table 3). Values normalized after a one-hour rest.

**Table 1:** Baseline characteristics.

		All	HIIT	MICT	p-value
		N = 117	N = 59	N = 58	
Gender	Women	23 (19.7)	8 (13.6)	15 (25.9)	0.108
Age	Years	56 (10.3)	55 (11.5)	57 (9.0)	0.293
BMI	kg/m^2^	28.8 (4.6)	28.3 (4.4)	29.3 (4.8)	0.244
Event	STEMI	71 (60.7)	35 (59.3)	36 (62.1)	0.850
	NSTE-ACS	46 (39.3)	24 (40.7)	22 (37.9)	
Culprit vessel	LAD	56 (47.8)	26 (44.1)	30 (51.7)	0.204
	LCX	13 (11.1)	8 (13.6)	5 (8.6)	
	RCA	34 (29.1)	20 (33.9)	14 (24.1)	
	Minor vessel	8 (6.8)	3 (5.1)	5 (8.6)	
	MINOCA	6 (5.1)	2 (3.4)	4 (6.9)	
Disease extent	Single vessel	92 (78.6)	48 (81.4)	44 (75.9)	0.871
	Double vessel	14 (12.0)	7 (11.9)	7 (12.1)	
	Multi vessel	5 (4.3)	2 (3.4)	3 (5.2)	
	Undetermined	6 (5.1)	2 (3.4)	4 (6.9)	
Risk factors	Arterial hypertension	86 (73.5)	46 (78.0)	40 (69.0)	0.301
	Diabetes mellitus	10 (8.5)	6 (10.2)	4 (6.9)	0.743
	Dyslipidaemia	75 (64.1)	38 (64.4)	37 (63.8)	1.000
	Family history	46 (39.3)	23 (39.0)	23 (39.7)	1.000
	Smoker	54 (46.2)	27 (45.8)	27 (46.6)	1.000
LVEF (%)		55.4 (6.6)	55.9 (6.0)	54.8 (7.1)	0.398
VO_2 peak_ (ml/kg/min)		22.6 (5.9)	23.1 (6.1)	22.0 (5.7)	0.338
Therapy	ASA	116 (99.1)	59 (100)	57 (98.3)	0.496
	Clopidogrel	14 (12.0)	8 (13.6)	6 (10.3)	0.853
	Prasugrel	25 (21.4)	12 (20.3)	13 (22.4)	
	Ticagrelor	78 (66.7)	39 (66.1)	39 (67.2)	
	ACE inhibitor/ARB	97 (82.9)	52 (88.1)	45 (77.6)	0.148
	Beta blocker	101 (86.3)	50 (84.7)	51 (87.9)	0.789
	Statin	117 (100)	59 (100)	59 (100)	1.000

*STEMI* ST-elevation myocardial infarction, *NSTE-ACS* non-STEMI acute coronary syndrome, *BMI* body mass index, *LAD* left descending coronary artery, *LCX* left circumflex coronary artery, *RCA* right coronary artery, *MINOCA* myocardial infarction with non-obstructed coronary artery, *LVEF* left ventricular ejection fraction, *ASA* acetylsalicylic acid, *ACE* angiotensin converting enzyme, *ARB* angiotensin receptor blocker.

Age, BMI, LVEF and VO_2 peak_ are reported as means (standard deviations); other variables are reported as number (proportions).

**Table 2:** Baseline characteristics: women compared to men.

		All	Women	Men	p-value
		N = 117	N = 23	N = 94	
Age	Years	56 (10.3)	57.5 (9.8)	55.8 (10.5)	0.473
BMI	kg/m^2^	28.8 (4.6)	27.5 (5.1)	29.1 (4.4)	0.151
Event	STEMI	71 (60.7)	12 (52.2)	59 (62.8)	0.354
	NSTE-ACS	46 (39.3)	11 (47.8)	35 (37.2)	
Culprit vessel	LAD	56 (47.8)	11 (47.7)	45 (47.9)	0.571
	LCX	13 (11.1)	2 (8.7)	11 (11.7)	
	RCA	34 (29.1)	6 (26.1)	28 (29.8)	
	Minor vessel	8 (6.8)	2 (8.7)	6 (6.4)	
	MINOCA	6 (5.1)	2 (8.7)	4 (4.3)	
Disease extent	Single vessel	92 (78.6)	19 (82.6)	73 (77.6)	0.357
	Double vessel	14 (12.0)	1 (4.3)	13 (13.8)	
	Multi vessel	5 (4.3)	1 (4.3)	4 (4.3)	
	Undetermined	6 (5.1)	2 (8.7)	4 (4.3)	
Risk factors	Arterial hypertension	86 (73.5)	13 (56.5)	73 (77.7)	0.063
	Diabetes mellitus	10 (8.5)	1 (4.3)	9 (9.6)	0.684
	Dyslipidaemia	75 (64.1)	14 (60.9)	61 (64.9)	0.809
	Family history	46 (39.3)	9 (39.1)	37 (39.4)	1.000
	Smoker	54 (46.2)	12 (52.2)	42 (44.7)	0.642
LVEF (%)		55.4 (6.6)	57.5 (7.4)	54.8 (6.2)	0.072
VO_2 peak_ (ml/kg/min)		22.6 (5.9)	17.5 (3.9)	23.7 (5.7)	< 0.001
Therapy	ASA	116 (99.1)	23 (100)	93 (98.9)	1.000
	Clopidogrel	14 (12.0)	4 (17.4)	10 (10.6)	0.214
	Prasugrel	25 (21.4)	2 (8.7)	23 (24.5)	
	Ticagrelor	78 (66.7)	17 (73.9)	61 (64.9)	
	ACE inhibitor/ARB	97 (82.9)	15 (65.2)	82 (87.2)	0.026
	Beta blocker	101 (86.3)	21 (91.3)	80 (85.1)	0.735
	Statin	117 (100)	23 (100)	94 (100)	1.000

*STEMI* ST-elevation myocardial infarction, *NSTE-ACS* non-STEMI acute coronary syndrome, *BMI* body mass index, *LAD* left descending coronary artery, *LCX* left circumflex coronary artery, *RCA* right coronary artery, *MINOCA* myocardial infarction with non-obstructed coronary artery, *LVEF* left ventricular ejection fraction, *ASA* acetylsalicylic acid, *ACE* angiotensin converting enzyme, ARB angiotensin receptor blocker.

Age, BMI, LVEF and VO_2 peak_ are reported as means (standard deviations); other variables are reported as number (proportions).

**Table 3:** Estimates and intervention effect for coagulation markers.

Parameter	Training group	T0: Baseline	T1: After exercise	T2: 1 h after exercise	Adjusted p value for time*group interaction
OCP* (abs sum)	*HIIT*	23.5 (22.1–25.0)	24.3 (22.8–25.7)	23.9 (22.4–25.4)	0.678
	*MICT*	23.9 (22.5–25.3)	24.8 (23.4–26.2)	24.3 (22.8–25.7)	
OHP* (abs sum)	*HIIT*	7.9 (7.2–8.7)	8.4 (7.6–9.2)	7.9 (7.2–8.7)	0.424
	*MICT*	8.4 (7.6–9.1)	8.8 (8.0–9.5)	8.4 (7.7–9.1)	
OFP* (%)	*HIIT*	66.3 (63.6–69.1)	65.3 (62.5–68.1)	67,1 (64.3–69.8)	0.370
	*MICT*	64.5 (61.8–67.2)	64.3 (61.6–67.0)	64.9 (62.3–67.6)	
Fibrinogen* (g/L)	*HIIT*	3.3 (3.1–3.5)	3.4 (3.2–3.6)	3.3 (3.1–3.4)	0.043
	*MICT*	3.6 (3.4–3.8)	3.7 (3.5–3.8)	3.5 (3.3–3.7)	
D-dimer* (µg/L)	*HIIT*	346 (278–414)	364 (296–432)	344 (269–421)	0.042
	*MICT*	427 (359–496)	444 (375–513)	486 (410–562)	
Von Willebrand* (%)	*HIIT*	151 (137–164)	158 (144–172)	153 (139–167)	0.104
	*MICT*	167 (153–180)	174 (160–188)	169 (155–183)	

*HIIT* high intensity interval training, *MICT* moderate intensity continuous training, *OCP* overall coagulation potential, *OFP* overall fibrinolytic potential, *OHP* overall haemostatic potential.

*Results displayed as estimates (95% confidence interval).

## Discussion

Our study has shown that a bout of exercise—either MICT or HIIT—causes a transient shift in haemostatic potential in patients with CAD. We detected a significant transient procoagulant effect—an acute increase in the immediate post-exercise period, followed by a recovery trend towards pre-exercise levels. Such haemostatic dynamic was consistently observed with both individual coagulation biomarkers and global haemostatic assays but was not significantly different between HIIT and MICT. Our findings suggest that exercise bout in patients with CAD is associated with an acute, transient increase in coagulation activity, irrespective of training intensity protocol.

Patients with CAD have an increased risk of atherothrombotic events, which may be partially explained by a procoagulant state^23–25,28,29^. Coagulation biomarkers and haemostatic potentials are increased in patients with CAD, suggesting the coagulation-fibrinolysis balance is shifted towards net coagulation^26,35^. In our study, increased baseline levels of OHP and OCP, and reduced baseline levels of OFP confirm previous reports of a procoagulant state in CAD^35,39^, which together with vascular damage (i.e., atherosclerosis) and blood stasis (i.e., atherothrombotic flow obstruction), contributes to a well-recognised prothrombotic state.

After an exercise bout, OHP, OCP, fibrinogen, D-dimer and von Willebrand factor levels increased, whereas OFP mildly decreased. Altogether, this haemostatic pattern reflects an exercise-induced shift towards coagulation, which is consistent with previous findings in healthy individuals^40–42^ and may provide one explanation for the acutely increased risk of atherothrombotic events during exercise^26,27^. During post-exercise recovery, coagulation biomarkers and OHP assays measurements returned to baseline pre-exercise levels, suggesting that the effect of exercise on procoagulation in patients with CAD is transient and may be limited to the first hour after exercise.

Possible explanations include stress-responses to excess, such as adrenergic stimulation, and endothelium-dependent and inflammation-mediated coagulation^43^. Acute bouts of exercise increase sympathetic activity, which, in turn, increases coagulation and fibrinolytic pathways yielding a net procoagulant effect^14^. Transient endothelial activation dysfunction and inflammation-oxidation hyperactivity during high-intensity exercise may promote platelet activity and procoagulant pathways^31^. Moreover, plasma volume depletion during exercise may result in haemoconcentration, which affects the measurement of individual coagulation biomarker levels but may also—in and of itself—drive the coagulation cascades^44^. Such haemostatic responses to exercise in healthy individuals have been interpreted as an evolutionary trait (protecting from potential bleeding during exercise-related injuries); in patients with CAD, however, the procoagulant pattern on top on increased baseline hypercoagulability may be regarded as unfavourable risk-associated derangement^4^.

Given the increasing popularity of HIIT protocols in cardiac rehabilitation programs^7,11–13,45^, we sought to compare the effects of HIIT and MICT on haemostasis in patients with CAD. Our findings suggest that MICT and HIIT exercise protocols do not elicit different haemostatic responses. A significant difference was appreciated only for D-dimer, which is an acute phase reactant and may reflect a non-specific stress-response to exercise intensity rather than a specific haemostatic derangement^46^. While previous studies in healthy individuals suggested that the coagulation balance may be related to exercise intensity, our results in patients with CAD suggest comparable haemostatic responses to either HIIT or MICT. One possible explanation pertains to the fact that baseline haemostasis in our CAD patient population was activated, with a negligible additional effect of exercise protocol (our study was powered to detect a moderate effect size of treatment /exercise training group, with the assumption that minor effect sizes do not represent a meaningful contribution to the haemostatic derangements). The other possible explanation pertains to exercise duration difference between exercise bout protocols. Our exercise protocols were designed to provide comparable energy expenditure in order to isolate exercise intensity pattern from exercise duration; by design, this resulted in shorter duration of HIIT as compared to MICT. Previous studies in healthy individuals suggest that both exercise intensity and duration affect the magnitude of haemostatic response^40^; our study in patients with CAD suggest that high-intensity exercise bouts of shorter energy expenditure-adjusted duration elicit comparable haemostatic responses to moderate-intensity bouts of longer energy expenditure-adjusted duration.

Our study population consisted predominantly of men, resembling the low number of women referred for cardiac rehabilitation programmes^47^. Multiple reasons are responsible for sex-specific disparities in rehabilitation referral, enrolment and completion. Physician bias may represent an important issue as well as low social support, lower functional capacity and lack of awareness^48,49^. Moreover, women are less likely to adhere and complete cardiac rehabilitation programmes, probably due to particular characteristics such as lower physical function and family issues^50^. Depression and anxiety are associated with lower levels of attendance to cardiac rehabilitation programmes and are more frequent in women than men^51^. Raising awareness among patients and health care providers about sex differences in cardiac rehabilitation, using systemic referral processes and providing more flexible times and programmes could help address this very important and still unresolved issue in cardiac rehabilitation.

Limitations of our study pertain primarily to its single centre design and the choice of surrogate endpoints. Firstly, single centre recruitment may incur in selection bias. Yet, the centre represents a large national referral centre, which gathers a varied patient population: given the baseline characteristics, risk factor profiles and baseline haemostatic measurements, our participants were comparable to other CAD populations undergoing cardiac rehabilitation and we believe that our results are generalizable to patients with CAD after a recent myocardial infarction. Secondly, surrogate endpoints, which provide insight into exercise-induced haemostatic derangements in patients with CAD, may not reflect clinically meaningful changes in cardiovascular health. Furthermore, measuring additional biomarkers, such as markers of endothelial function (e.g. VCAM-1 and ICAM-1) and inflammation (e.g. high sensitive CRP), would have provided more comprehensive understanding of the observed effects of exercise bout. Assessment of platelet function could provide a deeper understanding of the coagulation derangements in CAD patients during exercise, however given the longer turnover time of platelets compared to plasma coagulation markers^52^, we believe that acute changes after a bout of exercise would be better appreciated with coagulation and haemostasis assessment. Thirdly, patients on anticoagulation treatment were excluded due to the impact on haemostatic assays and increase in heterogeneity of results^36^. Given the low prevalence of anticoagulants among patients undergoing cardiac rehabilitation, we believe this had a minor impact on the generalisability and applicability of results^13^. Lastly, our study was limited to acute effects of MICT vs. HIIT exercise bout; future studies should try to address how an exercise training with structured repetitive MICT vs. HIIT may impact haemostasis in the long-term in patients with CAD.

Nonetheless, our study provides interesting and novel insights into the acute effects of two exercise protocols, which are routinely employed in cardiac rehabilitation programs for patients with CAD. With this knowledge our clinical practice should focus on advising patients after myocardial infarction to take part in organized cardiac rehabilitation. Rehabilitation protocols may include also HIIT, since it emerged as an equally safe protocol as MICT. However we should intensify the supervision during exercise and post-exercise period, knowing that the risk of cardiovascular events is increased during this time. As a future perspective, investigating the long-term effects of HIIT and MICT on coagulation (i.e., before and after completion of 8–12 weeks of exercise training program) would offer a deeper understanding of the pathophysiology and improve our informed selection of exercise protocols for CAD patients.

In conclusion our study has shown that a bout of exercise is associated with a transient increase in coagulation in patients with CAD, as appraised by changes in both global haemostatic assay (increased OHP and OCP, reduced OFP) and individual coagulation biomarkers (increased fibrinogen, D-dimer and von Willebrand factor levels). Conversely, our findings do not suggest a meaningful difference in the magnitude of effect on coagulation between HIIT and MICT after a bout of exercise.

## Methods

### Study population

Consecutive patients referred for cardiac rehabilitation program after acute myocardial infarction between December 2018 and March 2019 were screened for inclusion. We included patients 18–70 years of age with a recent myocardial infarction (within 90 days from inclusion). Patients with advanced heart failure (New York Heart Association class III and IV), uncontrolled arrhythmias, on anticoagulation treatment and pregnant women were excluded. The study was conducted in accordance with the Declaration of Helsinki (1964) and approved by the National Medical Ethics Committee (approval number 0120-77/2020/3).

### Study design

After signing the informed consent, patients were randomized into 2 groups (HIIT or MICT) with a 1:1 ratio using adaptive randomisation with a predefined fixed number of sealed envelopes and randomization concealment from the recruiting investigator^53^.

Based on validation studies for OHP and previous studies investigating the effect of exercise on coagulation and fibrilonlysis we estimated a 5–10% change in OHP^35,40,54^. Sample size calculation suggested at least 102 participants (61 per group) should be included to detect a moderate effect size (f = 0.3) with power of 0.80 at a significance level of 0.05 or less. Assuming a 10% drop-out rate, we aimed to include at least 110 participants.

Patients had three blood samples drawn from the cubital vein: before the intervention (T0), immediately after the intervention (T1) and after a one-hour resting period (T2). Blood was collected into 4.5 ml 0.109 M sodium citrate tubes. Citrated blood was then centrifuged for 20 min at 2000 × *g*, the extracted plasma was stored at − 70 °C for further analysis.

### Intervention

The intervention consisted of one session of rehabilitation exercise training on a stationary bike. The intervention was performed in a European validated centre for cardiac rehabilitation. All sessions were performed in the morning time (from 8 am to 2 pm) by a cardiovascular physical therapist, under the supervision of a cardiovascular nurse and a cardiologist, specialized in cardiac rehabilitation. Heart rate was monitored using a chest strap heart rate sensor (Polar Electro, USA) and a monitoring system (Sana Sprint Plus, Ergosana GMBH, Germany). The selected session was the first one in the cardiac rehabilitation programme. To ensure the same energy expense for both MICT and HIIT protocol, we followed the previously validated protocol of Rognmo et al.^12^. We calculated MICT and HIIT protocol length using the formula:

$$5 \,min\cdot 50\%{VO}_{2\, peak}+7\cdot \left(1.5 \,min\cdot 90\%{VO}_{2 \,peak}+3\,min\cdot 65\%{VO}_{2\, peak}\right)+5\,min\cdot 50\%{VO}_{2\, peak}=5\,min\cdot 50\%{VO}_{2 \,peak}+32\,min\cdot 75\%{VO}_{2 \,peak}+3\,min\cdot 50\%{VO}_{2\, peak}.$$

MICT protocol consisted of 5 min warm up at 50% HR_peak_, 32 min training at 75% HR_peak_ and 3 min cool-down at 50% HR_peak_. HIIT protocol consisted of 5 min warm-up at 50% HR_peak_, 7 cycles of 1.5 min at 90% HR_peak_ and 3 min at 65% HR_peak_ and 5 min cool-down at 50% HR_peak_. HR_peak_ was determined based on cardiopulmonary exercise testing at inclusion, performed before rehabilitation initiation. Cardiopulmonary exercise testing was performed on MTM 1500 Trackmaster (Schiller AG, Switzerland).During the intervention the fluid intake was ad libitum, with no restrictions, we recommended a healthy breakfast before rehabilitation sessions. Participants were recommended moderate aerobic physical activity during the cardiac rehabilitation programme, according to guidelines^1,2^.

### Measurements

We collected baseline data, cardiovascular risk factors. The researcher who collected the aforementioned data was blinded for the patients’ randomization group.

Fibrinogen (g/L), D-dimer (µg/L), von Willebrand factor (%), OHP, OCP and OFP were measured. Fibrinogen, D-dimer, and von Willebrand factor antigen were measured with the Dade^®^ Thrombin Reagent, Innovance D-dimer and vWFAg reagent kit, respectively (all Siemens Healthineers, Germany), on an automated coagulation analyser CS − 2500 (Sysmex, Japan) according to the manufacturer's instructions. Overall haemostatic potential (OHP, abs-sum), overall coagulation potential (OCP, abs-sum) and overall fibrinolytic potential (OFP, %) were determined as per previously described protocols^35,37^. OCP was determined by mixing 60 µL of plasma sample with 50 µL of buffer n°1 (1750 µL Tris–HCl buffer (pH = 7,4), 235 µL calcium chloride (CaCl_2_ 320 mmol/l) and 18 µL bovine thrombin (Sigma Chemical Company, St. Louis, USA)), OHP was determined by mixing 60 µL of plasma sample with buffer n°2 (1750 µL Tris–HCl buffer (pH = 7,4), 235 µL calcium chloride (CaCl_2_ 320 mmol/l), 18 µL bovine thrombin (Sigma Chemical Company, St. Louis, USA) and 15 µL recombinant tissue-type plasminogen activator (Actilyse 0.1 mg/ml, Boehringer Ingelheim, Germany)). The absorbance was measured for both samples at 405 nm in 1 min intervals for 40 min. Areas under the curve were constructed for OHP and OCP with the obtained measurements, OFP was calculated as the difference between the two aforementioned areas: OFP = [(OHP – OCP)/OCP] × 100 (%). Reference values (median with 5th and 95th percentile) for healthy individuals (n = 70) are as follows: OHP 7.9 (4.2–14.5) sum abs., OCP 11.8 (6.8–20.0) sum. abs., OFP 28 (16.5–52.0) %. Coefficients of variation of OHP and OCP are 8.7 and 3.7% for intra-assayrepeated measurements, and 5.1 and 4.2% for inter-assay repeated measurements, respectively, providing adequate reproducibility and requisite variety of the measurement methods.

### Statistical analysis

Baseline characteristics are expressed as mean (± standard deviation) for normally distributed continuous variables, as median (interquartile range) for non-normally distributed continuous variables and as frequency (%) for categorical variables. Between-group differences were assessed by t-test for normally distributed variables and proportions were compared using the χ^2^ test. Mixed effects linear models were constructed to assess the effects of exercise on haemostatic measures, with fixed effects. Between-group differences in change of haemostatic measures (dependent variables) were estimated using linear mixed effects models (accounting for repeated measurements in each patient). Patients were fitted as random effects, time (before exercise training, immediately after exercise training and one hour after exercise training), group (MICT vs. HIIT) and time*group interaction were assigned as fixed effects. P-values were adjusted with Bonferroni correction. In the context of randomised trials, a significant time*group interaction suggests a significant effect of group allocation on haemostatic parameter change over time (i.e., treatment effect)^55^. Results were expressed as estimates (95% confidence interval). A 2-tailed p < 0.05 was considered significant. Statistical analysis was carried out using SPSS Statistics version 23 (SPSS Inc, Chicago, USA) and R version 4.2.1, the R Foundation for Statistical Computing (Supplementary Table 1).

## Data Availability

The raw data supporting the conclusions of this article will be made available by the authors upon request.

## Supplementary Table 1

Supplementary Information.
