# Supplementary material for: Acute effects of high intensity interval training versus moderate intensity continuous training on haemostasis in patients with coronary artery disease
Source: Sci Rep. 2024 Jan 23;14:1963. doi: 10.1038/s41598-024-52521-6 (PMC10806221; doi:10.1038/s41598-024-52521-6)
Supplement: Supplementary file 1 — Supplementary Information. [file 41598_2024_52521_MOESM1_ESM.docx]

| **Parameter** | **Training group** | **T0: Baseline** | **T1: After exercise** | **T2: 1h after exercise** | **Adjusted p value for intervention** | **Adjusted p value for pairwise comparison** |
| --- | --- | --- | --- | --- | --- | --- |
| **OCP* (abs sum)** | *HIIT* | 23.5 (22.1–25.0) | 24.3 (22.8–25.7) | 23.9 (22.4–25.4) | 0.581 | T0 vs T1: 0.004 |
|  | *MICT* | 23.9 (22.5–25.3) | 24.8 (23.4–26.2) | 24.3 (22.8–25.7) |  | T0 vs T2: 0.675 |
| **OHP* (abs sum)** | *HIIT* | 7.9 (7.2–8.7) | 8.4 (7.6–9.2) | 7.9 (7.2–8.7) | 0.638 | T0 vs T1: 0.003 |
|  | *MICT* | 8.4 (7.6–9.1) | 8.8 (8.0–9.5) | 8.4 (7.7–9.1) |  | T0 vs T2: 1.000 |
| **OFP* (%)** | *HIIT* | 66.3 (63.6–69.1) | 65.3 (62.5–68.1) | 67,1 (64.3–69.8) | 0.694 | T0 vs T1: 0.375 |
|  | *MICT* | 64.5 (61.8–67.2) | 64.3 (61.6–67.0) | 64.9 (62.3–67.6) |  | T0 vs T2: 0.578 |
| **Fibrinogen* (g/L)** | *HIIT* | 3.3 (3.1–3.5) | 3.4 (3.2–3.6) | 3.3 (3.1–3.4) | 0.077 | T0 vs T1: < 0.001 |
|  | *MICT* | 3.6 (3.4–3.8) | 3.7 (3.5–3.8) | 3.5 (3.3–3.7) |  | T0 vs T2: 0.005 |
| **D-dimer* (µg/L)** | *HIIT* | 346 (278–414) | 364 (296–432) | 344 (269–421) | 0.055 | T0 vs T1: 0.011 |
|  | *MICT* | 427 (359–496) | 444 (375–513) | 486 (410–562) |  | T0 vs T2: 0.107 |
| **Von Willebrand* (%)** | *HIIT* | 151 (137–164) | 158 (144–172) | 153 (139–167) | 0.089 | T0 vs T1: <0.001 |
|  | *MICT* | 167 (153–180) | 174 (160–188) | 169 (155–183) |  | T0 vs T2: 0.220 |

**Supplementary Table – ANCOVA analysis for coagulation markers.** HIIT – high intensity interval training, MICT – moderate intensity continuous training, OCP – overall coagulation potential, OFP – overall fibrinolytic potential, OHP – overall haemostatic potential, * - results displayed as estimates (95% confidence interval).
